# The fruit extract of *Berberis crataegina* DC: exerts potent antioxidant activity and protects DNA integrity

**DOI:** 10.1186/s40199-015-0108-7

**Published:** 2015-04-17

**Authors:** Mohammad Charehsaz, Hande Sipahi, Engin Celep, Aylin Üstündağ, Özge Cemiloğlu Ülker, Yalçın Duydu, Ahmet Aydın, Erdem Yesilada

**Affiliations:** Faculty of Pharmacy, Department of Toxicology, Yeditepe University, 34755 Atasehir, Istanbul Turkey; Faculty of Pharmacy, Department of Pharmacognosy, Yeditepe University, 34755 Atasehir, Istanbul Turkey; Faculty of Pharmacy, Department of Toxicology, Ankara University, 06100 Tandoğan, Ankara Turkey

**Keywords:** *Berberis crataegina* DC, Folk medicine, Genotoxicity, Lipid peroxidation, Antioxidant

## Abstract

**Background:**

Dried fruits of *Berberis crataegina* (Berberidaceae) have been frequently consumed as food garniture in Turkish cuisine, while its fruit paste has been used to increase stamina and in particular to prevent from cardiovascular dysfunctions in Northeastern Black Sea region of Turkey. This study investigated this folkloric information in order to explain the claimed healing effects as well as to evaluate possible risks.

**Methods:**

Total phenolic, flavonoid and proanthocyanidin contents and antioxidant capacity of the methanolic fruit extract were evaluated through several *in vitro* assays. The cytotoxic and genotoxic effects of *B. crataegina* fruit extract were also assessed in both cervical cancer cell line (HeLa) and human peripheral blood lymphocytes.

**Results:**

The extract showed protective effects against ferric-induced oxidative stress and had a relatively good antioxidant activity. It also ameliorated the H_2_O_2_ mediated DNA damage in lymphocytes, suggesting the protective effect against oxidative DNA damage.

**Conclusion:**

The methanolic extract of *B. crataegina* fruits may be a potential antioxidant nutrient and also may exert a protective role against lipid peroxidation as well as oxidative DNA damage.

## Background

*Berberis* species (Berberidaceae) are known to exert spasmolytic, cholagogue, analgesic, anti-inflammatory, anxiolytic, antipsychotic, antidepressant, and potent scolicidal effects [1-3]. There are four naturally occurring species of *Berberis* in Turkey. Among these species, *B. crataegina* DC. and its hybrids are widely distributed and its black fruits are often consumed as food, and used as diuretic and expectorant. Moreover, the roots and root barks of this plant have been used in Turkish folk medicine against various ailments including jaundice, hemorrhoids, dysuria and as febrifuges in feverish conditions as well as tonic and appetizer [4,5]. On the other hand, the red fruits of another species, *B. vulgaris* L., have been used as a garniture in Persian food culture owing to its color and mellow taste [2,6], while the aqueous extract of barks is reported to be used to treat rheumatism and fever in Azerbaijan folk medicine [5] and Bulgaria [7].

Similar utilizations for the roots of several other *Berberis* species have also been reported elsewhere. In Uzbekistan, condensed aqueous extract of *B. oblonga* root is reported to be prescribed for the effective treatment of lumbago [8], while in Nepal, the condensed aqueous extract of *B. asiatica* Roxb. root barks is reported to be used orally against fever and in Pakistan powdered roots of *B. lycium* Royle are used orally with milk to treat rheumatic and muscular pains [5].

As summarized above, the underground parts of *Berberis* species have been used particularly against inflammatory disorders in worldwide traditional medicines, while the fruits of *Berberis* species have frequently been used as food, rather than for healing purposes. According to a recent report, the fruit paste of *B. crataegina* has been used to increase stamina and in particular to prevent from cardiovascular dysfunctions in Northeastern Black Sea region of Turkey [9].

Reactive oxygen species (ROS) and reactive nitrogen species (RNS), either generated exogenously or produced endogenously, are involved in a variety of biological phenomena such as pathogenesis of many diseases, mutation, carcinogenesis and aging [10,11]. Fortunately, there are several defense mechanisms for protection against these reactive molecules under physiological steady state conditions. Among these various defense mechanisms, the antioxidant system is extremely important due to its direct removal of pro-oxidants and maximum protection for biological sites [10]. Furthermore, it is important to protect the balance between oxidative stress and antioxidant defense mechanisms in order to prevent the adverse effects generated by oxidative stress [12]. Scientific investigations have demonstrated that phytochemicals are a great source for antioxidants, particularly the phenolic components, which have the structural requirements for free radical scavenging activity [1,12,13]. Ethanolic extracts of roots, twigs and leaves of *B. vulgaris* L. and also *B. croatica* Horvat showed some radical scavenging activity in correlation with the content of phenolic compounds [1]. In another study, Hanachi *et al*. [14] showed that *B. vulgaris* fruits exhibit antioxidant activity and have the ability to reduce the cell viability in human liver cancer cell line.

The present survey was aimed to evaluate the antioxidant effects of the fruit paste of *B. crataegina* in order to explain the claimed healing effects as well as to define the possible risks. For this purpose, the *in vitro* antioxidant potential of *B. crataegina* fruits extract was evaluated through several chemical and biochemical assays, including 2,2′-diphenyl-1-picrylhydrazyl (DPPH) radical-scavenging activity, superoxide anion radical scavenging activity, ferric reducing antioxidant power (FRAP), cupric reducing antioxidant capacity (CUPRAC), β-carotene bleaching, total antioxidant capacity (TOAC) and Trolox equivalent antioxidant capacity (TEAC). Also, the cytotoxic and genotoxic effects of *B. crataegina* fruits extract were investigated in both human cervival cancer cell line (HeLa) and human peripheral blood lymphocytes. In addition to these tests, the total contents of phenolic, flavonoid and proanthocyanidin of the fruits were also determined.

## Material and methods

### Preparation of fruit extract

The dried *B. crataegina* fruits (2 kg) were provided from Bayburt province (Turkey) and authenticated by one of the authors (E.Y.). A voucher specimen was deposited in the Herbarium of Faculty of Pharmacy, Yeditepe University (YEF 10018). The fruits were washed, mashed in a blender with 500 mL of warm distilled water and freeze-dried (Christ Alpha 2-4 LD). Then 200 g of dried fruit sample were extracted twice with 50 mL of 80% methanol (Sigma) at 45°C for 4 hours with continuous stirring. The combined extract was filtered through a filter paper and then was evaporated to dryness under reduced pressure. Finally, the sticky residue was dissolved in distilled water and freeze-dried [BCFE] (yield, 9.2%).

### Phytochemical screening of the extract

The method previously described was used for determination of total phenolic content [15]. Accordingly, 20 μL of properly diluted BCFE sample were mixed with Folin–Ciocalteu reagent (Sigma) and Na_2_CO_3_ (20%) (Riedel de Hean). Then, samples were incubated at 45°C for 30 minutes. At the end of this period, the absorbance was measured at 765 nm by UV-Vis spectrophotometer (Thermo, Evolution 300). Results were expressed as mg gallic acid equivalents (GAE) per gram of dried extract.

For the determination of total flavonoid content, 500 μL of properly diluted BCFE sample were mixed with 10% AlCl_3_ (Merck) and 1 M sodium acetate (Riedel de Hean). Following the incubation period (30 minutes at room temperature), the absorbance was recorded at 415 nm. Results were expressed as mg quercetin equivalents (QE) per g of dried extract [13].

The total proanthocyanidin content of the BCFE sample was measured by adding of 2.5 mL of vanillin (1%) (Fluka) and 2.5 mL of 9 M HCl (Sigma) in methanol to properly diluted extracts. After incubating at 30°C for 20 min, the absorbance was measured at 500 nm. Total proanthocyanidin content of samples was expressed as mg epigallocatechingallate equivalents (EGCG-E) per g of dry extract [13].

### Measurement of *in vitro* antioxidant activity

DPPH radical-scavenging activity, superoxide anion radical scavenging activity, FRAP, CUPRAC, β-carotene bleaching, TOAC and TEAC were measured spectrophotometrically by the methods previously described by Celep *et al*. [13].

### *In vitro* cytotoxicity and genotoxicity studies

#### Treatment of human peripheral lymphocytes

The donor was a 30 years old woman (non-smoker) and her health status was completely compatible with the WHO guideline on the blood donor selection criteria.

##### Treatment procedure I

Lymphocytes were isolated from the whole blood by using LeucoSep® (greiner bio-one) centrifuge tubes according to the instruction manual provided by the manufacturer. Briefly, the anticoagulated blood sample was poured into the Leucosep tube and centrifuged for 10 min (1000xg). The lymphocytes appeared at the interface between the plasma (top layer) and separation medium (Ficoll, 1.077 g/ml). This enriched cell fraction was harvested by means of a Pasteur pipette, washed with 10 ml of phosphate-buffered saline (PBS), and centrifuged for 10 min at 250 x g. Afterwards, 50 μL aliquots of the cell (lymphocyte) suspension were dispensed into micro centrifuge tubes (1x10^4^-2x10^4^ cells/50 μL). 1 mL aliquots of BCFE (0.001, 0.005, 0.01, 0.05, 0.1, 0.2, 0.5, 1, 2, and 4 mg/mL) were added into related micro-centrifuge tubes and incubated for 2 hours. Two micro-centrifuge tubes were allocated as positive control and treated with 50 μM and 100 μM H_2_O_2_ at the last 5 minutes of the incubation period. The micro-centrifuge tubes were centrifuged at 250 g and the supernatants were discarded. The DNA damage in lymphocytes was identified by using the alkaline comet assay.

##### Treatment procedure II

The same procedure described above was applied with the following exception; the micro-centrifuge tubes containing BCFE were treated with 50 μM H_2_O_2_ at the last 5 minutes of the 2 hours incubation period. Afterwards, the procedure proceeded with centrifugation as was described above. The DNA repair in lymphocytes was identified by using the alkaline comet assay.

### Comet assay

#### Preparation of the cells

The same comet assay procedure was used for both HeLa cells and lymphocytes. The details of the procedure were formerly described [16,17]. Briefly; 50 μL aliquots of the cell suspension (1x10^4^ - 2x10^4^ HeLa cells or lymphocytes/50 μL) were mixed with 100 μL of low melting point agarose (0.5% LMA) (Sigma) and dispensed onto the microscope slides previously coated with normal melting point agarose (1% NMA) (Sigma). The suspension spread by using a coverslip and left on an ice-cold flat tray for 5 minutes. After removal of the cover slip, the slides were immersed in lysing solution.

#### Lysing

The slides were immersed into formerly prepared cold (4°C) lysing solution containing 2.5 M NaCl (Sigma), 100 mM Na_2_EDTA (Sigma), 10 mM Tris (Sigma), 1% sodium sarcosinate (Sigma), (pH 10) with 1% Triton-X 100 (Sigma) and 10% DMSO (Sigma) and left there for 1 hour, afterwards the slides were removed from the lysing solution and drained.

#### Electrophoresis

The slides were placed in horizontal gel electrophoresis tank containing the electrophoresis solution (1 mM Na_2_EDTA and 300 mM NaOH, pH 13). Electrophoresis was then conducted for 20 min by applying an electric current of 25 V/300 mA to allow damaged DNA to migrate from the nucleus toward the anode.

*Neutralization:* The slides were then drained, placed on tray and washed with three changes of neutralization buffer (0.4 M Tris, pH 7.5) for 5 minutes each. The slides were left to drain before staining.

#### Staining

The slides were stained with 50 μL (20 μg/mL) ethidium bromide (Sigma) and covered with a coverslip. Then the slides were viewed using a fluorescence microscope (Leica DM1000) equipped with an excitation filter of 515-560 nm. A single scorer randomly selected and captured 100 cells using the Perceptive Instruments COMET Assay IV analysis system. Tail % intensity was selected as the image analysis parameter. Two slides were prepared for each single sample. The results are given as the mean of both slides.

### Trypan blue viability test

The cell suspension (lymphocytes) was diluted (1:1) with 0.4% trypan blue solution (Sigma) and carefully filled the hemocytometer (Improved Neubauer) chamber. The viable (unstained) and non-viable cells (blue) were counted under a microscope.

#### Treatment of HeLa cells

The HeLa cell line was cultured as a monolayer in an appropriate tissue culture flask at 37°C with 5% CO_2_ in F12 HAM containing 10% heat inactivated fetal bovine serum (Sigma), 50 μg/mL penicillin (Biological Industries) and 50 μg/mL streptomycin (Biological Industries). When cells approach confluence, they were removed from the flask by trypsinization. After counting the cells, the culture is seeded into two 24-well plates (5x10^4^ cells/well) and incubated at 37°C with 5% CO_2_ for 24 hours. Afterwards, 1 mL aliquots of BCFE (0.001, 0.005, 0.01, 0.05, 0.1, 0.2, 0.5, 1, 2, and 4 mg/mL) were added into related wells and incubated for 2 hours. Two wells were allocated as positive control and treated with 50 μM and 100 μM H_2_O_2_ (Merck) at the last 5 minutes of the incubation period. The cells were removed from the wells by trypsinization and the supernatant was discarded after centrifugation at 2500 rpm for 5 minutes. The DNA damage in HeLa cells was identified by using the alkaline comet assay.

In another set of 24-well plates, the HeLa cells were exposed 24 hours to the equal concentrations of BCFE by using the same procedure described above.

### Neutral Red Uptake (NRU) cytotoxicity test

The HeLa cells were cultured as described above then seeded into a 96-well microtiter plate (1x10^4^ cells/well) and incubated at 37°C with 5% CO_2_ for 24 hours. Afterwards, the culture medium was removed and the cells were treated with 100 μl treatment medium containing either 8 concentrations of BCFE or the positive control (sodium dodecyl sulfate, SDS) (Merck). After 24 hours of treatment period, medium were removed and the cells were washed with 150 μl PBS (Thermo). Thereafter, PBS was aspirated and the cells were incubated in 100 μl of neutral red medium (Sigma) for additional 3 hours. After this final incubation period, neutral red medium were discarded and washed with 150 μl PBS. Finally 150 μl ethanol (Sigma)/acetic acid (Sigma) (1% glacial acetic acid, 50% ethanol, %49 H_2_O) solution were added to all wells and the 96-well plate was shaken for 10 minutes in a micro plate shaker. The absorption of the colored solution was measured at 540 nm by a micro plate reader (SpectraMax 190) and the related IC_50_ values were computed [18].

### Statistical evaluation

All of the results are expressed as the mean ± SD. For *in vivo* data, the differences between the groups were evaluated with Kruskal-Wallis analysis of variance and comparisons between two independent groups were made with the Mann-Whitney *U*-test. For the data of the comet assay, the analysis of variance (ANOVA) was used to determine whether there are any significant differences between the means of the groups. The Dunnett test was used as part of the ANOVA test to determine whether means were different from mean of the control. All statistical tests were performed with SPSS for Windows Release 11. p < 0.05 was considered statistically significant.

## Results and discussion

### Phytochemical screening of the extract

Phenolic compounds are the major class of bioactive components. Previous reports have shown that the fruits of various *Berberis* species are rich in polyphenolic constituents and eventually fruit extracts have shown to possess potent free radical-scavenging activity [19-21] due to the polyphenolic compound’s ability to act as hydrogen donors, reducing agents and radical scavengers [1].

Flavonoids and proanthocyanidins are one of the major polyphenolic constituents of plants because of the radical scavenging ability conferred by their hydroxyl groups at various positions; particularly of an ortho-dihydroxy structure in their B ring [15]. A previous study revealed that polyphenols can also prevent oxidative stress-mediated DNA damage [22].

Limited numbers of studies have previously been reported the phenolic contents of the fruits of *Berberis* species, i.e. *B. vulgaris,* [23] while no report have been found on the polyphenolics of B. *crataegina* fruits in a reference survey. In present study, total phenolics, flavonoids and proanthocyanidins contents of 80% methanolic extracts of BCFE were evaluated and results were expressed as mg gallic acid, quercetin and epigallocathechingallate equivalents, respectively (Table 1).

**Table 1:** **The total phenolic, flavonoid and proanthocyanidin content of 80% MeOH extract of** ***B. crataegina*** **fruit (BCFE)** ^***A***^

	**Total phenolic content (mg GAE/g extract)** ^**B**^	**Total flavonoid content (mg QE/g extract)** ^**C**^	**Total proanthocyanidin content (mg EGCG-E/g extract)** ^**D**^
BCFE	53.51 ± 3.62	27.42 ± 1.34	548 ± 19.7

^A^Results were expressed as the mean of triplicates ± standard deviation (S.D.).

^B^Total phenolic content was expressed as mg gallic acid equivalents (GAE) in 1 g dried extract ± S.D.

^C^Total flavonoid content was expressed as mg quercetin equivalents (QE) in 1 g dried extract ± S.D.

^D^Total proanthocyanidin content was expressed as mg epigallocathecin gallate equivalents (EGCG-E) in 1 g dried extract ± S.D.

### *In vitro* antioxidant activity

Since free radicals are one of the main causes of oxidative stress, the ability of BCFE on free radical scavenging was assessed by DPPH, superoxide radical scavenging and TEAC tests in this study [15]. As given in Table 2, DPPH radical scavenging activity of BCFE was about 30% of reference substance BHT and superoxide radical scavenging activity was about 2% of reference substance gallic acid. These results indicate that BCFE does not have a good activity against superoxide radicals but have a relatively good activity against other stable radicals like DPPH. On the other hand, Fe^3+^ and Cu^2+^ involve in the formation of free radicals and the reduction of ferric as well as cupric ions indicate another mechanism of antioxidant potential [13]. As shown in Table 2, ferric reducing power of BCFE was higher than that of reference substance BHT and copper reducing activity was about the 5.6% of ascorbic acid. These results indicate that BCFE can prevent ferric induced oxidative stress efficiently but not for copper. As an indicator of the prevention of lipid peroxidation, β-carotene bleaching assay demonstrated that BCFE to possess about 80% of activity of reference substance BHT, suggesting a good activity against lipid peroxidation (Table 2). Furthermore, total antioxidant capacity of BCFE was about 8.6% of ascorbic acid and 20% of Trolox, indicating a relatively good antioxidant activity (Table 2).

**Table 2:** ***In vitro*** **antioxidant activities of 80% MeOH extract of** ***B. crataegina*** **fruit (BCFE)** ^**A**^

	**DPPH radical scavenging activity** ^**B**^	**Superoxide radical scavenging activity** ^**C**^	**FRAP** ^**D**^	**CUPRAC** ^**E**^	**β-carotene bleaching assay** ^**F**^	**TOAC** ^**G**^	**TEAC** ^**H**^
**BCFE**	405 ± 11.6	9.04 ± 0.92	0.76 ± 0.03	56.3 ± 0.17	77 ± 2.2	86.69 ± 4.62	198 ± 5.6
BHT*	133 ± 6.4		3.02 ± 0.07		96 ± 2.6		
**Gallic acid**		0.18 ± 0.01					

^A^Results were expressed as the averages of triplicates ± standard deviation (S.D.), ^B^IC_50_, expressed in μg/mL, ^C^IC_50_, expressed in mg/mL, ^D^Ferric reducing antioxidant power was expressed as mM FeSO_4_ equivalents in 1 g material, ^E^Copper reducing antioxidant capacity was expressed as mg ascorbic acid equivalents in 1 g material, ^F^The results of β-carotene bleaching assay was given as % in 1 mg/mL extract or reference compound, ^G^Total antioxidant capacity was expressed as mg ascorbic acid equivalents in 1 g material, ^H^Trolox equivalent antioxidant capacity was expressed as μM Trolox equivalent in 1 g material, *Butylated hydroxy toluene.

#### In vitro evaluation of genotoxicity and cytotoxicity

**Figure 1:**
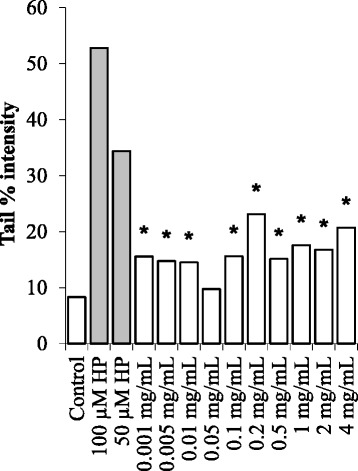
The DNA integrity of HeLa cells treated with increasing concentrations of BCFE for 2 hours. *The tail % intensity was significantly increased when compared with the control (p < 0.05). Black bars representing the positive controls.

**Figure 2:**
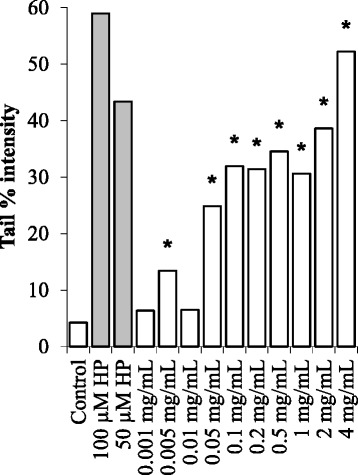
The DNA integrity of HeLa cells treated with increasing concentrations of BCFE for 24 hours. *The tail % intensity was significantly increased when compared with the control (p < 0.05). Black bars representing the positive controls.

The genotoxic potential of the BCFE in HeLa cells was tested at concentrations lower than its IC_50_ value. Since the DNA damaging potential of H_2_O_2_ is well known and has been reported previously in several published studies [16,17], H_2_O_2_ (50 and 100 μM) was used as a positive control. The HeLa cells were treated with increasing concentrations of BCFE in order to assess its effect on the DNA integrity. The DNA integrity of HeLa cells was identified by using the comet assay and expressed in terms of tail % intensity. As shown in Figure 1, the mean tail % intensity was significantly increased in each tested concentrations with the exception of 0.05 mg/mL (p < 0.05) under the treatment duration of 2 hours. These results indicate a negative effect of BCFE on the DNA integrity of HeLa cells. On the other hand, the mean tail % intensity values were completely changed under the treatment duration of 24 hours. The mean tail % intensity value was sharply increased at the concentration of 0.05 mg/mL and even the mean tail % intensity value of the positive control (50 μM H_2_O_2_) was exceeded at the concentration of 4 mg/mL as shown in Figure 2. However, the mean tail % intensity values determined for the concentrations of 0.001 and 0.01 mg/mL were decreased to the control level. Apparently, the damaged DNA was repaired in 24 hour. These results indicate an adaptive cellular response in HeLa cells at these two concentrations of BCFE.

**Figure 3:**
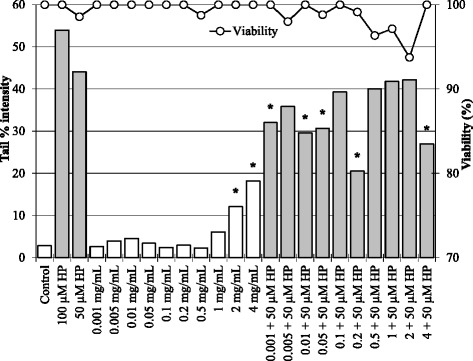
Dose-dependent effects of BCFE on the DNA integrity of human peripheral blood lymphocytes. The H_2_O_2_ (50 and 100 μM) treated lymphocytes are the controls for the combined treatments (black bars). The white and black bars represent the mean tail % intensity values determined after the treatment period of 2 hours. *Significantly different from the related control. The viability of lymphocytes varied between 93.75% and 100% within the tested concentrations.

The viability of lymphocytes varied between 93.75% and 100% within the tested concentrations are shown in Figure 3. The genotoxic effect of BCFE was determined also in human peripheral blood lymphocytes. The DNA damage started to occur at 2 mg/mL with a significantly increased (p < 0.05) mean tail % intensity value. The mean tail % intensity values at the lower concentrations were not statistically different from the mean tail % intensity value of the control. In other words, the DNA integrity of lymphocytes was not affected by BCFE at the concentration of 1 mg/mL or lower.

Interestingly, the results obtained in HeLa cells and lymphocytes were not comparable to each other. Although, HeLa cells and lymphocytes were treated with the same concentrations of BCFE for the same period of time (2 hours), HeLa cells were more vulnerable to the components in BCFE as shown in Figure 1. Apparently, human peripheral blood lymphocytes are more resistant to the in BCFE.

The influence of H_2_O_2_ (50 μM) on the DNA integrity of lymphocytes treated with increasing concentrations of BCFE was investigated within the study. Accordingly, the DNA damage generated by 50 μM H_2_O_2_ was significantly decreased in lymphocytes previously treated with BCFE at concentrations of 0.001, 0.01, 0.05, 0.2, and 4 mg/mL (Figure 3). BCFE has clearly ameliorate the H_2_O_2_ mediated DNA damage in lymphocytes at the above mentioned concentrations. In other words, BCFE might be protective against oxidative DNA damage. This protective effect might be a reflection of the induced DNA repair or the antioxidant capacity of polyphenolic compounds in BCFE [21]. On the other hand, the DNA damage of lymphocytes was significantly induced with BCFE at 4 mg/mL. In spite of this genotoxic effect at 4 mg/mL, the H_2_O_2_ (50 μM) induced oxidative DNA damage was significantly reduced at the same extract concentration.

**Figure 4:**
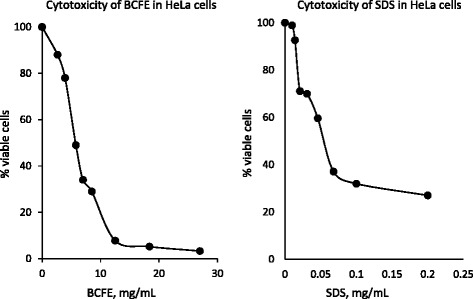
The cytotoxic effects of BCFE and SDS (positive control). The IC_50_ values of BCFE and SDS were 4.98 mg/mL and 0.055 mg/mL, respectively.

The results obtained in NRU cytotoxicity tests were used to identify the IC_50_ value of the BCFE in HeLa cells. Accordingly, the computed IC_50_ values of BCFE and SDS (positive control) were 4.98 mg/mL and 0.055 mg/mL, respectively (Figure 4).

## Conclusions

In conclusion, the present study has revealed that BCFE possesses potent total antioxidant activity, scavenging stable radicals like DPPH, prevent ferric induced oxidative stress and has a good activity against lipid peroxidation. Also, the BCFE can be a potent protective nutrient against oxidative DNA damage. However, it should be considered that the results of *in vitro* antioxidant assays may sometimes conflict with the results obtained from *in vivo* models. Therefore, in order to ascertain the role of dietary antioxidants fully, *in vivo* tests are incredibly necessary.
